# TH1/TH2 Cytokine profile in relapsing-remitting multiple sclerosis patients treated with Glatiramer acetate or Natalizumab

**DOI:** 10.1186/1471-2377-12-95

**Published:** 2012-09-18

**Authors:** Celia Oreja-Guevara, Jaime Ramos-Cejudo, Luiz Stark Aroeira, Beatriz Chamorro, Exuperio Diez-Tejedor

**Affiliations:** 1 Department of Neurology, Neuroimmunology and Multiple Sclerosis Unit, University Hospital La Paz, Madrid, Spain; 2 Neuroscience Research Laboratory. Health Research Institute (IdiPAZ), University Hospital La Paz, Autónoma University of Madrid, Madrid, Spain; 3 Department of Neurology, IdISSC, University Hospital San Carlos, Madrid, Spain

**Keywords:** Multiple sclerosis, Th2, Th1, Cytokines, Glatiramer acetate, Natalizumab

## Abstract

**Background:**

The balance between T helper cells Th2- and Th1-related cytokines plays a key role in multiple sclerosis (MS). A shift from a Th1 towards a Th2 cytokine profile could have a beneficial effect on the clinical course of the disease. The objective of this study was to assess Th2/Th1 cytokine profile in relapsing-remitting MS (RRMS) patients receiving an immunosuppressive treatment with natalizumab (NAT), or an immunomodulatory treatment with glatiramer acetate (GA) after one year of treatment.

**Methods:**

This was an observational cross-sectional study. All consecutive patients diagnosed with RRMS who had received GA or NAT for 12 months were included in the study. We determined serum levels of Th1 and Th2 cytokines (interleukin [IL]-1a, IL-1b, IL-2, IL-4, IL-5, IL-6, IL-8, IL-10, IL-12p70, IL-13, monocyte chemotactic protein [MCP]-1, tumor-necrosis factor [TNF]-α, interferon [IFN]-γ and granulocyte macrophage colony stimulating factor [GM-CSF]) by flow cytometry. Th2/Th1 bias was defined based on the ratio of IL-4, IL-5, IL-6 or IL-10 Th2 cytokines and proinflammatory INF-γ or TNF-α Th1 cytokines.

**Results:**

Eleven patients under treatment with NAT and 12 patients treated with GA were evaluated. RRMS patients treated with NAT showed significantly higher levels of IL-6 (p < 0.05), MCP-1 (p < 0.01), and GM-CSF (p < 0.05) compared to GA patients after one year of treatment. A trend for increasing of IL-12p70, IL-1b, TNF- α and IFN- γ levels was also found in patients receiving NAT compared to GA patients. IL-4/IFN-γ, IFN-γ/TNF-α and IL-10/IFN-γ ratios as markers of Th2/Th1 ratio were significantly elevated in GA patients compared to those receiving NAT (p < 0.05).

**Conclusion:**

In conclusion, our findings suggest that GA promotes a superior Th2-biased anti-inflammatory response as compared with NAT in the systemic circulation of RRMS patients. Future studies with larger cohorts will determine whether this immune Th2 shift in GA patients is associated with a beneficial effect on disease outcome.

## Background

Multiple sclerosis (MS) is an inflammatory demyelinating disease of the central nervous system (CNS). T helper (Th) cells appear to play a pivotal role in the autoreactive immune response of the CNS in MS, primarily characterized by inflammation and demyielination [1,2]. Cytokines are key factors in the regulation of inflammatory responses and may reflect the disease process in MS [3,4]. Th cells can be divided into subtypes based on the characteristic cytokine secretion patterns and their effector functions. While Th2-related cytokines such as interleukin IL-4, IL-10, or IL-5 have been associated with inflammation reduction and improvement of symptoms in MS patients, Th1 cytokines such as interferon-gamma (INF-γ) and tumor necrosis factor-alpha (TNF-α) have been shown to increase inflammation, therefore leading to disease progression and worsening of symptoms [5-10]. Th2 and Th1 cytokines can cross-inhibit each other and the progression of disease may depend on the balance between both types of cytokines. Induction of Th1 cytokines toward Th2 could achieve the suppression of undesirable autoimmunity and have a beneficial effect on the clinical course of the disease [11]. In this scenario, effective treatments should induce a shift toward Th2 cytokine anti-inflammatory response.

Treatment of relapsing-remitting MS (RRMS) with glatiramer acetate (GA) (Copaxone®) reduces frequency of relapses and the appearance of new lesions in gadolinium enhanced magnetic resonance imaging (MRI) [12,13]. As other immunomodulatory treatments, GA seems to interfere with the Th1/Th2 balance by promoting a shift from the Th1 to the Th2 anti-inflammatory cytokine pathway [14-22]. Natalizumab (NAT) (Tysabri®), a humanized monoclonal antibody against very late activation antigen (VLA)-4 on leukocytes, has demonstrated to reduce the relapse rate by about 70% in RRMS patients [23,24]. The widely proposed mode of action of NAT is based on the reduction of transmigration of leukocytes into the CNS [25,26]. However, other immunological effects may also be operative accounting for changes of some Th1/Th2 cytokines levels in plasma of RRMS patients treated with NAT [27,28].

In the present study, we aimed to evaluate the patterns of Th2/Th1 cytokines in RRMS patients receiving an immunosuppressive treatment with NAT, or an immunomodulatory treatment with GA after one year of treatment.

## Methods

The present study was a cross-sectional, observational study conducted at a University Hospital. Written informed consent was obtained from all patients before they were included in the study. The study was approved by the Ethics Committee of the University Hospital La Paz, Madrid.

### Patients

All consecutive patients diagnosed with RRMS who had received GA (Copaxone®) or NAT (Tysabri®) for 12 months were included in the study. All patients fulfilled the McDonald criteria for RRMS [29]. Patients underwent a complete neurological examination to quantify patients’ disability by the Kutzke's Expanded Disability Status Scale (EDSS) every 6 months. All patients treated with NAT had been previously treated with immunomodulatory treatments (GA or β-interferon; minimum wash-out period of 1 month). The GA-treated patients were *naïve* for previous treatments.

### Cytokine analysis

Briefly, blood samples were collected from patients after 12 months of treatment and serum was obtained by centrifugation and stored at −80°C until cytokine determination. All samples were collected before each drug administration.

Serum levels of Th1- and Th2-related, MS-relevant cytokines (interleukin [IL]-1a, IL-1b, IL-2, IL-4, IL-5, IL-6, IL-8, IL-10, IL-12p70, IL-13, monocyte chemotactic protein [MCP]-1, tumor-necrosis factor [TNF]-α, interferon [IFN]-γ and granulocyte macrophage colony stimulating factor [GM-CSF]) were determined by flow cytometry (FacsCalibur, BD Biosciense, CA, USA) using CBA Flex Set kit (BD Bioscience, Bedford, MA, USA) following manufacturer’s instructions. CBA Flex Set analysis was performed using FCAP array v1.0 software (Soft Flow Inc., USA). Protein values were converted to NIBSC/WHO protein standards for further comparisons.

### Statistical analysis

Median serum cytokines levels between GA-treated patients and those receiving NAT were compared using StudentÂ´s *t*-test.

Th2/Th1 ratio was defined based on the ratio of IL-4, IL-5, IL-6 or IL-10 Th2-related cytokines and proinflammatory INF-γ or TNF-α Th1-related cytokines. The median Th2/Th1 ratio was calculated for each group. The non-parametric Mann-Whitney U test was performed in order to compare both groups using GraphPad Prism 5.0 software (San Diego, CA, USA), and *t*-test under log transformation of Th2/Th1 ratio is presented.

## Results

### Patient characteristics

A total of 23 RRMM patients treated with GA or NAT for 12 months were included in the study. Eleven patients under treatment with NAT and 12 patients treated with GA were evaluated.

The study population was comprised by 9 females in each group (82% and 75% in NAT and GA group, respectively) and 2 and 3 males (18% and 25%) in NAT and GA group, respectively. The mean age was 37.73 ± 7.24 and 37.67 ± 10.58 years old for NAT and GA-treated patients correspondingly.

Mean disease duration for patients treated with NAT and GA was 7.90 ± 3.42 and 4.25 ± 2.70 years, respectively. The mean EDSS score at the start of treatment ranged from 0 to 6.50 for all patients, with a mean of 4.05 ± 1.67 in NAT-treated patients and 1.67 ± 1.77 in those receiving GA (p = 0.005). After 12 months of treatment, mean EDSS decreased by 0.2 and 0.1 in the NAT and GA groups, respectively.

### Th1/Th2 cytokine profile

**Figure 1:**
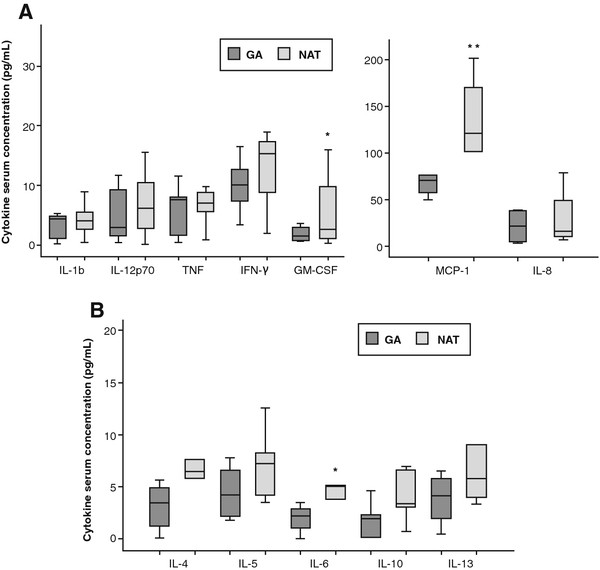
**Serum levels of cytokines after one year of treatment with GA or NAT in RRMS patients.** Cytokines are grouped as Th1-related (**A**) or Th2-related (**B**). Cytokines were determined by Flow cytometry. All concentrations are given in pg/mL and were converted to international WHO/MIBBSC standards. Data are shown as median and interquartile ranges. NAT-treated patients showed significantly higher levels of proinflammatory cytokines MCP-1, and GM-CSF compared to those patients treated with GA. IL-6 Th2-related cytokine was significantly higher in NAT group. *p < 0.05; **p < 0.01. GA = glatiramer acetate; NAT = natalizumab.

Overall, patients receiving NAT showed significantly higher levels of proinflammatory cytokines IL-6 (p < 0.05), MCP-1 (p < 0.01) and GM-CSF (p < 0.05) compared to GA-treated patients after one year of treatment. Median levels of IL-6, MCP-1 and GM-CSF in NAT patients were 5.21 pg/L (3.80-6.36), 121.23 pg/L (95.86-157.68) and 4.05 pg/L (1.38-12.19), respectively, whereas GA patients showed median levels of 2.88 pg/L (1.05-5.75), 65.41 pg/L (41.70-75.89) and 1.56 pg/L (0.68-3.23) for IL-6, MCP-1 and GM-CSF, respectively. Moreover, serum levels of Th1-related cytokines IL-12p70, IL-1b, TNF-α, and IFN-γ showed a clear tendency to be higher in patients receiving NAT as compared to GA patients (Figure 1).

**Figure 2:**
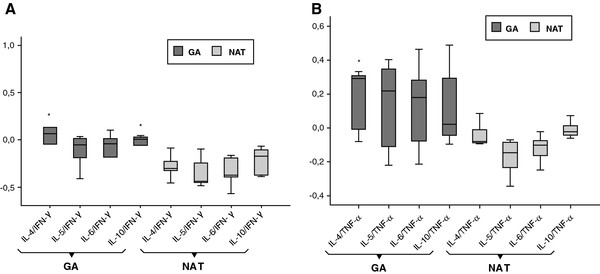
**Th2/Th1 ratio in GA and NAT patients.** Comparative Th2/Th1 ratios considering anti-inflammatory IL-4, IL-5, IL-6, IL-10 cytokines and pro-inflammatory cytokine INF-γ (**A**) or TNF-α (**B**). Box and whiskers plots showing median and interquartile ranges are presented. IL-4/IFN-γ, IL-4/TNF-α and IL-10/IFN-γ ratio as markers of Th2/Th1 ratio are significantly higher for GA patients compared to patients receiving NAT. *p < 0.05. GA = glatiramer acetate; NAT = natalizumab.

IL-4/IFN-γ, IL-4/TNF-α and IL-10/IFN-γ ratio as markers of Th2/Th1 ratio were significantly higher for GA-treated patients as compared to those receiving NAT after one year of treatment (p < 0.05) (Figure 2).

## Discussion

The results of the present study suggest a Th2/Th1 balance shift in favour of a Th2 cytokine profile on GA-treated patients, while NAT causes a predominant Th1-biased response. This superior anti-inflammatory shift of GA seems to be mainly mediated by raising IL-4 and IL-10 levels which could lead to a down-regulation of Th1 cytokine secretion. Accordingly, the enhancement of circulating IL-4 and IL-10 and the subsequent detrimental effect on IFN-γ and TNF-α seen in GA-treated RRMS patients may play a protective role from inflammatory response that could affect the clinical course of disease in these patients. In this regard, a stabilization of disability score was found in both NAT and GA patients after one year of treatment. The potential clinical implications of immune response in GA-treated patients have been previously assessed [30-32]. Indeed, Valenzuela et al. [31] reported an increased IL-4/IFN-γ ratio to be associated with a favourable clinical outcome in a study of 36 RRMS patients treated with GA. However, the available findings suggesting the potential association between specific cytokine patterns and clinical response to GA were controversial primarily due to the short follow-up period. A recent study with a longer follow-up period of 3 years has demonstrated that IL-2 + IFN-γ/IL-10 + IL-4 ratio was significantly elevated in those patients with RRMS that suffered from relapses and progressing brain atrophy, suggesting that a specific pattern of Th2/Th1 cytokines may predict clinical response to GA therapy [33]. Moreover, this study suggests that the quotient IL-4 + IL-10/IL-2 + IFN-γ could be a promising parameter to identify patients associated with a highly beneficial response to GA therapy. However, although follow-up data over 3 years were available in this study, the sample size was relatively small to draw firm conclusions. Consequently, further studies including larger cohorts of patients will be required to validate that clinical and immune response correlate in patients treated with GA. Additionally, the mechanisms underlying the relation between cytokine response and clinical outcome in GA treated patients remain as a matter of debate.

The results of the present study show that patients treated with NAT exhibit higher levels of circulating proinflammatory cytokines and chemokines than those treated with GA. These findings are in agreement with previous studies where NAT treatment has been associated with an increased expression of proinflammatory cytokines in peripheral blood mononuclear cells [34,35]. Accordingly, an increase in activated leukocytes producing proinflammatory cytokines has been found in peripheral blood of NAT-treated patients [34,36]. Although it is not clear, these findings could probably be due to the inhibition of transmigration of lymphocytes into CNS, resulting in sequestration of activated T cells in the peripheral circulation [36]. The prolonged T-cell activation could result in decreased local immunosurveillance, reactivation of latent viral infections or opportunistic CNS infections, as evidenced by the rare but severe occurrence of progressive multifocal leukoencephalopathy caused by JC virus in NAT-treated patients [37]. Recent evidence has suggested that NAT seem to exert its beneficial effect without affecting regulatory T cell function [28]. Interestingly, NAT therapy has been associated with an increase in some pro-inflammatory and anti-inflammatory cytokines within the first 2 months of therapy, whereas relevant cytokines for MS such as IL-2, IL-7, or IL-1β have been found to be increased after one year of treatment, suggesting different immunological mechanisms [28]. The changes in the Th1/Th2 paradigm do not appear to be applicable to explain the beneficial effect of NAT. The increase of circulating Th1 cytokines could be related to a “rebound” effect that led to the development of new and enlarging T2 lesions previously seen in cohorts of patients discontinuing NAT due to safety issues related to this therapy, particularly regarding PML [38]. This finding has led to the concern that cessation of NAT might promote a worsening of MS disease by increasing inflammatory activity. The most likely explanation is that short exposure to NAT (e.g., 2 infusions) results in blockade of migration and accumulation of activated lymphocytes in the periphery that retain their capacity to cause CNS disease [39].

The main limitations of this study include the small sample size and a one-point measurement of cytokine patterns. However, despite these limitations, our findings are potentially interesting given that to our knowledge, this is the first study to compare the Th1/Th2 bias between GA and NAT treated RRMS patients.

## Conclusion

In summary, GA seems to modulate Th1/Th2 balance in the systemic circulation with a shift toward the anti-inflammatory Th2 profile response in RRMS patients. This effect may have a beneficial effect on disease activity in these patients. Further studies including larger cohorts of patients and a larger follow-up are needed in order to establish whether this immune Th2 shift in GA patients correlates with a favourable clinical response. NAT seems to exert is beneficial effect through different mechanisms than immunomodulators such as GA.

## Competing interests

E. Diez-Tejedor has collaborated as clinical advisor investigator in clinical trials and as speaker with the following companies: Astra-Zeneca, Bayer, Bristol-Myers Squibb,Boehringer Ingelheim, Cellerix, Ferrer Grupo, Knoll, Lilly, Parke-Davis, Pfizer, Sanofi-Synthelabo, Servier, UCB Pharma, Uriach, EVER Neuro Pharma. C. Oreja- Guevara has collaborated as speaker and in clinical trials with Biogen Idec, Merck Serono, Teva, Sanofi, Bayer-Schering and Novartis.

## Authors’ contributions

All authors had full access to all the data in the study and take responsibility for the integrity of the data and the accuracy of the data analysis. Study concept and design: COG. Acquisition of data: COG, BC. Analysis and interpretation of data: COG, JRC, LSA. Drafting of the manuscript: COG, JRC, EDT. Statistical analysis: COG, JRC. All authors read and approved the final manuscript.

## Pre-publication history

The pre-publication history for this paper can be accessed here:

http://www.biomedcentral.com/1471-2377/12/95/prepub
